# *Boletus edulis* Nitrite Reductase Reduces Nitrite Content of Pickles and Mitigates Intoxication in Nitrite-intoxicated Mice

**DOI:** 10.1038/srep14907

**Published:** 2015-10-08

**Authors:** Weiwei Zhang, Guoting Tian, Shanshan Feng, Jack Ho Wong, Yongchang Zhao, Xiao Chen, Hexiang Wang, Tzi Bun Ng

**Affiliations:** 1 State Key Laboratory for Agrobiotechnology and Department of Microbiology, China Agricultural University, Beijing 100193, China; 2 Institute of Biotechnology and Germplasmic Resource, Yunnan Academy of Agricultural Science, Kunming 650223, P.R. China; 3 School of Biomedical Sciences, Faculty of Medicine, The Chinese University of Hong Kong, Shatin, New Territories, Hong Kong, China

## Abstract

Pickles are popular in China and exhibits health-promoting effects. However, nitrite produced during fermentation adversely affects health due to formation of methemoglobin and conversion to carcinogenic nitrosamine. Fruiting bodies of the mushroom *Boletus edulis* were capable of inhibiting nitrite production during pickle fermentation. A 90-kDa nitrite reductase (NiR), demonstrating peptide sequence homology to fungal nitrite reductase, was isolated from *B. edulis* fruiting bodies. The optimum temperature and pH of the enzyme was 45 °C and 6.8, respectively. *B. edulis* NiR was capable of prolonging the lifespan of nitrite-intoxicated mice, indicating that it had the action of an antidote. The enzyme could also eliminate nitrite from blood after intragastric administration of sodium nitrite, and after packaging into capsule, this nitrite-eliminating activity could persist for at least 120 minutes thus avoiding immediate gastric degradation. *B. edulis* NiR represents the first nitrite reductase purified from mushrooms and may facilitate subsequent applications.

Pickles (picked vegetables), a traditional dish favored by many Chinese, are mildly salted and lactic acid-fermented vegetables in China. During pickle fermentation, vegetables are immersed in a 6–8% salt solution and left to undergo lactic acid fermentation for 4–10 days at different temperatures. *Lactobacillus* produced or added during this fermented process is known to be beneficial to human health^1^. These bacteria contribute to the homeostasis of the microbial environment in the intestinal tract and maintain intestinal health. Metabolic activities of *Lactobacillus* during fermentation produces lactic acid, folic acid and vitamins which boost the human immune system. In addition, pickles exert the health-promoting effects of producing weight loss and preventing myocardial infarction and atherosclerosis. On the other hand, nitrite which deleteriously affects health is inevitably produced as a result of fermentation during the course of pickle production. Nitrite is a strong oxidizing agent. It oxidizes ferrous hemoglobin which transports oxygen in the blood to ferric hemoglobin. As a consequence, hemoglobin loses its oxygen transport capability leading to hypoxia^2^,^3^,^4^,^5^. In the acidic environment produced by gastric acid, nitrite is converted into carcinogenic nitrosamine^6^,^7^. Hence, the presence of nitrite in pickles is a bottleneck which limits further development of the pickle industry. More and more attention is drawn to the problem of the presence of nitrite in pickles, now that there is increased awareness of people about food safety. The National Food Safety Department in China has developed a standard for regulating the levels of nitrite and nitrate in vegetables and their products. According to the national food safety standard in China regarding the maximum allowable level of contaminant in food (GB2762-2005), the nitrite content in preserved vegetables should not exceed 20 mg/kg sample.

The mushroom *Boletus edulis* is well known for being delicious and nutritious. The crispy fruiting bodies can be sliced and eaten raw. When the mushroom is used to prepare soup, it gives off a pleasant fragrant smell. *B. edulis* is also nutritious because of the abundance of amino acids, polysaccharides and vitamins. Reports on its antitumor and antioxidant activities have recently been published^8^,^9^,^10^,^11^,^12^. In our investigation, it was disclosed that the fruiting bodies of *B. edulis* were capable of eliciting a marked reduction of the nitrite content in pickles. Hence we added the fruiting bodies during the process of pickle production and investigated the effect on nitrite content in pickles. This provides a new approach and technology for lowering the nitrite content in pickles.

## Results

### *B. edulis* exerted an effect on nitrite content of pickles

**Figure 1:**
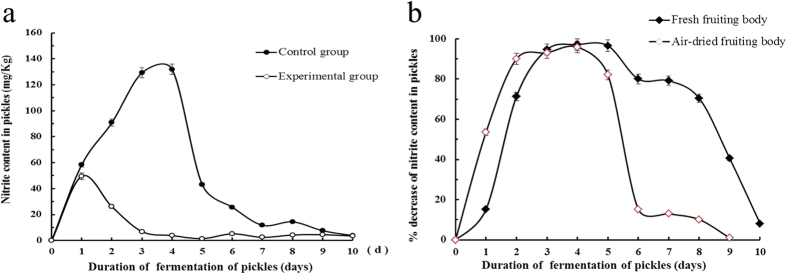
The effect of *Boletus edulis* in decreasing nitrite content during pickles fermention. (**a**) Differences in changes of nitrite content between pickles in control group (with no addition of mushroom) and experimental group (with addition of mushroom) during the period of fermentation. (**b**) Percent decrease of nitrite content in pickles of experimental group relative to pickles in control group during the period of fermentation. Results are presented as mean ± SD (n = 3).

To test the effect of fresh *B. edulis* fruiting bodies on the nitrite content of pickles, the fruiting bodies were added to the pickles (1:1, w-w), and the nitrite level was determined daily throughout the fermentation period, in comparison with pickles without prior exposure to fruiting bodies. A decline in nitrite content in the experimental pickles relative to the untreated control pickles was obvious on the second day of fermentation, and the decrease became even more pronounced on the third to the fifth day. The maximum (97.1%) reduction of nitrite content occurred on the 4^th^ day of fermentation. Thereafter, the difference between the control and experimental groups became attenuated. On the 10^th^ day, there was no discernible difference between the control and experimental groups (Fig. 1). The overall trends of variations in the nitrite content during the fermentation process were consistent, being high initially followed by a decrement. This is in keeping with other reports. In the early stage of fermentation, the intestinal coliform bacteria and *Pseudomonas* under aerobic conditions will produce nitrite using nitrate in the new materials, accounting for a rise in the nitrite level at the initial stages of fermentation. As the oxygen in the pickles pot decreases and the pH falls due to the metabolic activities of *Lactobacillus*, the environment is no longer conducive to the survival of nitrate-producing bacteria. Anaerobic bacteria such as nitrite-producing bacteria begin to proliferate massively and there is an accumulation of organic acids and vitamins beneficial to human health. As the nitrite-producing bacteria decrease in number and *Lactobacillus* causes nitrite to break down, the amount of nitrite gradually declines. Consequently the content of nitrite exhibits a trend of an initial rise and a subsequent fall, forming a conspicuous “nitrite peak”^13^. Furthermore, we tested the addition of various doses ranging from 10% to 50% *B. edulis* fresh fruiting bodies to pickles, and found that introduction of 30% fruit bodies was able to decrease the nitrite concentration by 92%.

An experiment was designed to test the effects of addition of (i) 50 g *B. edulis* fruiting bodies which had been air-dried at room temperature and (ii) 50 g oven-dried (60 °C) *B. edulis* fruiting bodies on changes in the nitrite levels in pickles. The results revealed that *B. edulis* fruiting bodies which had been oven-dried were devoid of any effect. In contrast, the air-dried fruiting bodies manifested a nitrite-lowering action which was slightly less potent than those produced by fresh and frozen fruiting bodies. It can be observed from the results on air-dried samples (Fig. 1b) that on the 4^th^ day of fermentation, the decline in nitrite content in the experimental group (mushroom-treated group) relative to the control group (with no mushroom treatment) reached a maximum, with a percent decline of 95.9%. As the duration of fermentation was prolonged, there was a steady diminution in the nitrite content of pickles. In the later phase of fermentation, the percent drop in nitrite content gradually became smaller.

*B. edulis* fruiting bodies, regardless of whether they are fresh, previously stored frozen or air-dried at room temperature, were all capable of lowering nitrite level in pickles, though the effect produced by air-dried fruiting bodies was slightly inferior to the effects of fresh and frozen fruiting bodies. Fruiting bodies dried in an 60 °C oven were totally devoid of nitrite lowering activity. The results indicate that the nitrite- lowering activity was retained after air-drying at room temperature but abolished after exposure for a long time to a high temperature, signifying that the active substance is a thermolabile substance. The identity of the substance being NiR explains its thermolability.

### Isolation of NiR from *Boletus edulis*

**Figure 2:**
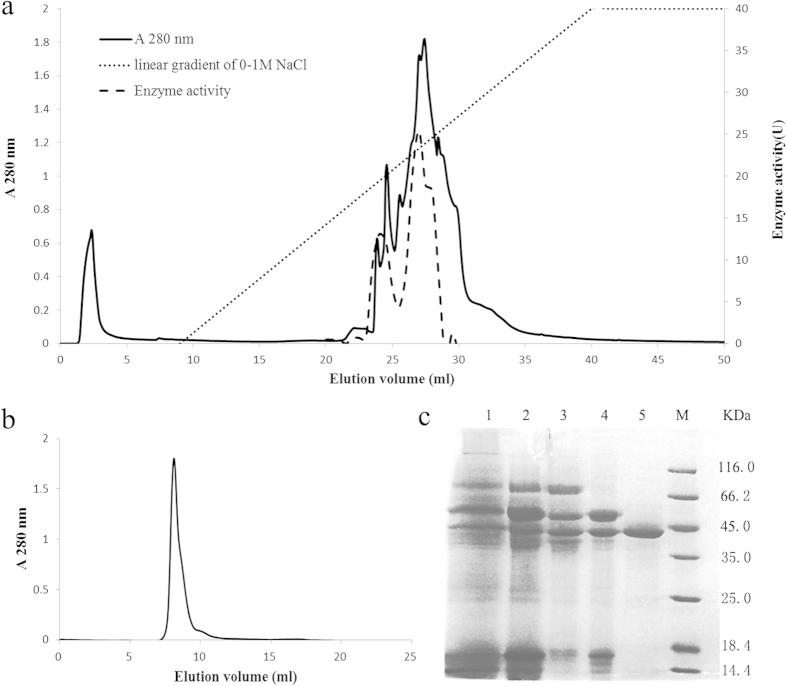
Purification of NiR. (**a**) Ion exchange chromatography of fraction Q1 (previously adsorbed on Q-Sepharose) on Mono Q. The column was initially equilibrated with 10 mM NaOAc-HOAc (pH 5.2) buffer and eluted with a linear NaCl concentration (0–1 M) in 10 mM NaOAc-HOAc buffer (pH 5.2). (**b**) Profile of elution of *Boletus edulis* fruiting body extract from Superdex 75 column (Fraction size = 0.8 ml). (**c**) SDS-PAGE of fractions of each purification procedure. Lane 1: crude sample. Lane 2: fraction after ammonium sulfate precipitation (25%–65%). Lane3: protein after chromatography on MonoQ 4.6/100 PE. Lane 4: enzyme after gel filtration on FPLC superdex-75. Lane 5: standard protein markers.

A 15.5-fold purification of the enzyme was achieved after the fruiting body extract had been subjected to (NH_4_)_2_SO_4_ precipitation, anion exchange chromatography on SP-Sepharose, Q-Sepharose and Mono Q, and molecular sieve chromatography (Table 1). Nitrite reductase activity in the fruiting body extract was not adsorbed on the SP-Sepharose cation exchanger. The first peak (first dotted activity peak), enriched in nitrite reductase activity eluted from Mono Q (Fig. 2a), was applied to Superdex 75. A single peak (~90 kD) was obtained (Fig. 2b). It demonstrated a single band with a molecular mass of 45 kDa after SDS-PAGE (Fig. 2c). According to the FPLC-gel filtration results, we suggest that this NiR is homodimeric and consists of two subunits each possessing a molecular mass of 45 kDa. It is worth mentioning that the second peak (second activity peak) with nitrite reductase activity eluted from Mono Q (Fig. 2a) displayed a band with a molecular mass of 50 kDa, but the band was not completely purified and required further investigations. Due to the limited stability of the enzyme at room temperature, the loss of enzyme activity was slightly higher leading to a purification fold which was not remarkable. LTQ mass spectroscopy analysis of the 45-kDa protein yielded 9 peptides with sequence complete matching in bacterial database and limited homology to fungi (Table 2 shows the alignment with NiR of some species). A sequence of the 15 N-terminal amino acids was also obtained and used for Basic Local Alignment Search Tool (BLAST) search in the database of *B. edulis* gonome which was accessible at Joint Genome Institute (JGI). The BLAST output was only one hit which presents a scaffold without annotation. This CDS sequence was translated to a sequence of 434 amino acids by DNAMAN. However, when using BLAST to search the 434- amino acid sequence in NCBI, the high score hits were almost all hypothetical or predicted protein. This result may probably due to the inadequacy of fungal, especially macrofungal genome database and the low homology of this enzyme.

**Figure 3:**
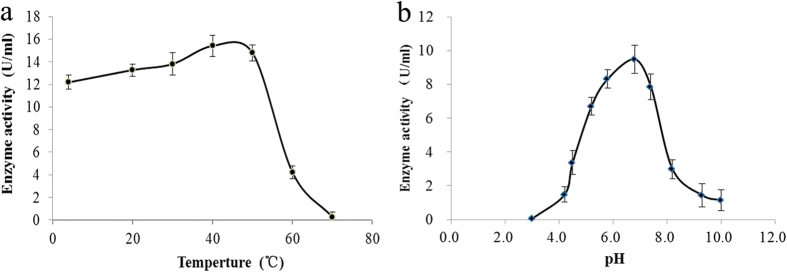
Properties of *Boletus edulis* nitrite reductase. (**a**) optimum temperature; (**b**) optimum pH. Results are presented as mean ± SD (n = 3).

We also determined the physiochemical characteristics of isolated NiR. The optimum temperature of the enzyme was 45 °C while the optimum pH was 6.8 (Fig. 3). Enzyme activity was extremely low at pH values below 4.5. Difficulty would be experienced when using it as a nitrite-eliminating agent *in vivo* since the gastric fluid is acidic. The kinetic parameters of this NiR were determined using different concentrations of nitrite. The Km value for nitrite was 19.73 mM and Vmax value was 2.95 U/mg of protein.

### NiR acted as an antidote for intoxicated mice

The nitrite-intoxicated mice exhibited purple lips, purple eyes and frailty. Excessive intake of nitrite could be life-threatening. To test if the NiR had nitrite reducing activity *in vivo*, different treatments of mice were conducted. The survival time and death rate of the mice after different treatments are shown in Table 3.

Mice intoxicated with the low dose of nitrite (0.015 g/100 g) would not be poisoned to death while those treated with the intermediate (0.025 g/100 g) or high dose (0.036 g/100 g) of nitrite would. However, when treated concurrently with the mushroom extract, the symptoms of intoxication could be relieved which is obvious in the group treated with the intermediate group dose (groups 2, 3 and 4). Mice treated with the intermediate dosage of nitrite concomitantly with low (34.2 U) and high (57 U) dosage of NiR extracts could live a significantly longer life than control (group 2), and some of them could survive the poisoning. Increasing the dosage of NiR extracts brought about better detoxication. The toxic symptoms of the surviving mice disappeared in about 5 hours. These results manifested that *B. edulis* NiR was capable of prolonging the lifespan of intoxicated mice indicating that it had the action of an antidote.

### The isolated NiR could eliminate nitrite in blood

**Figure 4:**
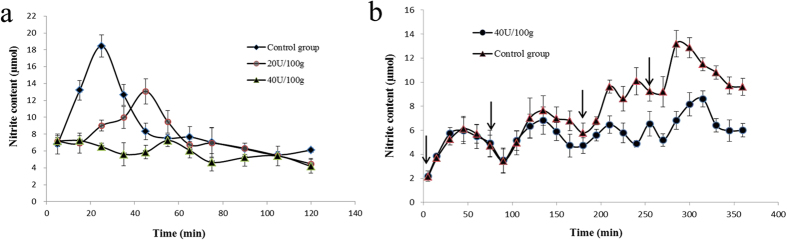
Changes of blood nitrite level in rats. (**a**) rats receiving NiR extracts; (**b**) rats receiving capsules containing the nitrite reductase. Arrows represent time point at which nitrite was administered. Results are presented as mean ± SD (n = 12).

Based on the ratio of conversion between the dosage for human and dosage for rats, the dose of NaNO_2_ lethal to rats was 420 mg/kg. NaNO_2_ was introduced by intragastric administration into rats at the dose of 7 mg/100 g body weight to raise the nitrite level. Blood nitrite level was determined after administration of different amounts of the NiR. The results are shown in Fig. 4a. It can be seen that there was a peak in the control rats which reached a maximum 25 minutes after intragastric administration of NaNO_2_. Then the peak gradually became smaller in size. In the experimental group that received *B. edulis* NiR, there was a shift in the peak which attained a maximum 45 minutes after intragastric administration. This shift and the smaller peak were most likely due to the elimination of nitrite by the NiR in *B. edulis*. As the dosage of NiR administered was elevated, the peak of blood nitrite level disappeared. Reduction of nitrite took place before 100 minutes even with the higher dose of NiR, which can be attributed to gastric degradation.

For a longer duration of action of nitrite reductase *in vivo*, we packaged this NiR into capsules to protect the enzyme from gastric degradation. As shown in Fig. 4b, the nitrite content appeared to form a waveform peak as gavaging was repeated and the peak was higher in the control group. In the first 105 min, there was no difference between the control and experiment groups. But from 120 min onward, the difference in nitrite content between the two groups gradually became more pronounced. The same experiment was conducted using *B. edulis* extracts with the same NiR activity replacing the capsules, and it turned out that reduction of nitrite only occurred before 100 minutes (data not shown). It can be speculated that these enzymes, with the ‘cloth’ protected, escaped from pepsin and superacid in the stomach and reached the intestine. The capsule dissolved in succus entericus and released the NiR, Thus the nitrite reduction time was postponed. Furthermore, the nitrite reduction period continued from 120 min to 360 min, which was substantially longer than 100 min in the control group. The results suggested that the NiR was more stable in the intestine than the stomach, which was consistent with the optimum pH for the enzyme. We suggest that *B. edulis*, with NiR activity, is capable of reducing nitrite *in vivo*. When NiR is encapsulated, the activity could be significantly prolonged.

## Discussion

Nitrite is a natural constituent of the human diet and an approved food additive present not only in pickles, but also in many preserved foods and fermented foods. The relevance of nitrate/nitrite to human health has been studied by numerous researchers. Nitrate and nitrite can be partially converted into NO which could induce vasodilation thus reducing the risk regarding cardiovascular disease^14^. On the other hand, nitrite was considered to be hazardous to health^15^,^16^,^17^. Nitrate and nitrite are not carcinogenic per se in some cases but under endogenous conditions N-nitroso compounds are formed which may lead to an increased cancer risk in humans. Effects on health, either beneficial or detrimental, are dose dependent and need a reliable assessment^18^. In Korea, kimchi is a daily favorite dish. However, nitrite is present in kimchi and may contribute to the high incidence of gastric carcinogenesis among Southwest Koreans^19^. Meat products, like sausage and ham, also contain nitrite because of its use as a preservative and flavor enhancement^16^. Cheese, which is popular worldwide, is also recognized as a nitrite-rich food. Above all, excessive nitrite consumption has become a focus of scientists’ concern. To date, agents capable of eliminating nitrite have been extracted from green tea^20^, vitamin C^21^, sodium erythorbate^22^ and vitamin E^23^,^24^. Various attempts have been made to reduce the concentration of nitrite in food. During pickle fermentation, *Lactobacillus* was found to contribute to the reduction of the nitrite in fermented food^13^,^25^. Antioxidants, such as ascorbic acid and erythorbic acid, are often used to inhibit the formation of nitrosamines in meat products^26^. Nevertheless, the acid produced would increase markedly when *Lactobacillus* is added instead of natural fermentation, in which case the taste would be affected^25^. The antioxidants have their drawbacks including low efficiency and high cost. *B. edulis* is a nutritious and delicious edible mushroom, hence, safety can be guaranteed. The fruiting bodies, as a material fermented with vegetables, have the potential use of reducing nitrite in pickles. The fruiting bodies, either fresh or dried at room temperature, can significantly reduce the nitrite level. The use of this mushroom can ensure retention of the unique flavor of pickles and depression of the nitrite level. The effect is striking, with a maximal (97.1%) reduction of nitrite content. The NiR activity could remain viable for at least two years for fresh fruiting bodies stored at −20 °C, and could be maintained for one year in fruiting bodies dried at room temperature. It deserves mention that the delicious fruiting bodies of *B. edulis* can be eaten raw thus circumventing the denaturing action of high temperatures. The results of the present investigation provide a new technology and approach conducive to the promotion of the pickle industry and development of associated industries. It also safeguards the health of the vast population of pickle consumers. We further analyzed the NiR activity in the mushroom and isolated it as a 90-kDa protein with two subunits. The meager homology to other species indicates that it is a novel enzyme. This NiR activity is relatively stable below 50 °C and has an optimum pH at 6.8. Results of the *in vivo* experiment support the abovementioned results. The nitrite-intoxicated mice receiving intragastric administrations of NiR had a longer lifespan than the control group. The concentration of nitrite in blood in the nitrite-intoxicated rat can also be lowered by the enzyme. Due to the low yield of the purified enzyme, it is difficult to obtain sufficient purified enzyme for the animal experiment, so crude enzyme was used instead. However, a small scale animal experiment using an equivalent amount of purified NiR yielded similar results and thus it can be speculated that the nitrite eliminating action is attributed to the enzyme. Nevertheless, it is unclear that if there are other components synergistic with the NiR in undermining the nitrite content. There are few reports on purified macrofungal NiRs. When *B. edulis* NiR is compared with other purified NiRs in several aspects (Supplementary Table S2), we did not find features common with other species.

As mentioned above, the fact that so many flavored foods contain nitrite prompted us to find a safe and convenient way to safeguard our health. Cold *B. edulis* fruiting bodies may be a good choice. As the data presented in this paper, 40 U NiR, equivalent to 0.8 g fresh *B. edulis* fruiting bodies, could significantly suppress the blood nitrite level in rats. According to the dose relationship, about 40 g fresh fruiting bodies or 4–5 g dried fruiting bodies would be appropriate for human intake when consuming nitrite-rich food. The aforementioned results suggest that *B. edulis* NiR is the first nitrite reductase purified from mushrooms which can alleviate nitrite toxicity. Thus the mushroom enzyme may find a variety of applications.

## Methods

All experimental protocols were approved by the University Safety Office and Animal Experimentation Ethics Committee at The Chinese University of Hong Kong and China Agricultural University. All the methods were carried out in accordance with the approved guidelines of the Animal Care and Use Committee of The Chinese University of Hong Kong and China Agricultural University.

### Materials

Fresh *B. edulis* fruiting bodies were purchased from a vendor of edible mild mushrooms in Yunnan, China and kept at −20 °C. SP-Sepharose, Q-Sepharose, Mono Q 4.6/100 PE column and Superdex 75 10/300 GL prepacked column were obtained from GE Healthcare (China). Sprague-Dawley rats and ICR mice were provided by Xinglong Experimental Animal Breeding Factory in Haidian District, Beijing. Reagents were of the finest grade available.

### Pickles fermentation and nitrite measurement

The brine was prepared by adding 300 g edible salt, 150 g sugar, 10 g pepper, 10 g chili, 5 g cinnamon, and 5 g star anise into water and boiling for 5 minutes. Then it was allowed to cool down. Radishes and *B. edulis* fruiting bodies were washed, dried, and cut into strips. Sliced ginger and garlic were also prepared. A 1000 g mixture of radishes and mushrooms (1:1 ratio by weight) was decanted into a 1 L pot. The pot was not more than 3/5 full. The control group had only radishes and no mushrooms. The cooled brine was added until it covered the vegetables. The pot was covered with a lid, and some water was added into the groove of the lid to maintain an oxygen-free environment. The fermentation was allowed to continue for ten days at room temperature. The nitrite content of pickles was determined using a colorimetric nitrite assay described in GB 5009.33–2010. Five grams of pickles were taken out and crushed each day during the fermentation period, and deproteinated and defatted by precipitation with 5 ml Zn(CH3COO)_2_ and 5 ml K_4_[Fe(CN)_6_] followed by filtration. Then 2 ml aminobenzene sulfonic acid (4 g/L) and 1 ml N-(1-naphthyl) ethylenediamine dihydrochloride solution(2 g/L) were added sequentially to 40 ml filtrates, diluted to 50 ml and mixed. The mixture was then kept for 15 min and the A_538 nm_ was read. The content of nitrite was read from the standard curve.

### Purification of nitrite reductase (NiR)

Fruiting bodies of *B. edulis* (500 g) were thawed, followed by addition of 500 mL deionized water, and extracted using a homogenizer, and then left to extract overnight at 4 °C. The mixture was centrifuged at 9000 rpm at 4 °C to obtain a crude preparation of NiR. The crude enzyme preparation was subjected to precipitation using 25% ammonium sulfate. The supernatant obtained after centrifugation was collected and then subjected to precipitation using 65% ammonium sulfate. The precipitate was dissolved, dialyzed and then chromatographed on an SP-Sepharose cation exchange column using phosphate buffer at pH 5.8. The unadsorbed fraction which contained NiR activity was loaded on a Q-Sepharose anion exchange column and eluted using a linear 0–1 M NaCl gradient in the phosphate buffer at pH5.8. The fraction containing NiR activity was dialyzed extensively and then chromatographed on a Mono Q ion exchange column using phosphate buffer (pH5.8). The active fraction collected during elution with a linear 0–1 M NaCl gradient was dialyzed and then chromatographed on a Superdex 75 gel filtration column. All chromatographic procedures were carried out at 25 °C.

### Assay of NiR

The assay used was a slight modification of the method described by Martínez-Espinosa *et al.*^27^. The reaction system contained 125 μL 0.1 M Tris-HCl buffer (pH 7.0), 15 μL 0.1 M NaCl, 12.5 μL 0.1 M NaNO_2_ , 7.5 μL 0.1 M methyl viologen, 50 μL enzyme solution, and 40 μL 0.1 M Na_2_S_2_O_4_ (freshly prepared in 0.1 M NH_4_HCO_3_ solution). Na_2_S_2_O_4_ was added to start the reaction. The reaction was allowed to proceed for 20 minutes in a 50 °C water bath, and then terminated by vigorous agitation. For determination of nitrite, a 10-μL aliquot of the reaction mixture was taken and diluted to 1 mL with distilled water, aminobenzene sulfonic acid solution (4 g/L, 40 μL) was added, well mixed, and the mixture was allowed to stand for 3–5 minutes for diazonium coupling to occur^28^. N-(1-naphthyl) ethylenediamine dihydrochloride solution (2 g/L, 20 μL)was then added, well mixed, and allowed to stand for 15 minutes to facilitate color development. OD_538 nm_ was then read^29^. The determination of the enzyme activity was based on a calculation of the amount of sodium nitrite broken down in the reaction i.e. the initial amount of sodium nitrite minus the residual amount of sodium nitrite after reaction for a certain period of time. A unit of enzyme activity was defined as the amount of enzyme required for reducing 1 μg nitrite per minute.

### Determination of molecular mass and amino acid sequence of isolated NiR

Sodium dodecyl sulfate polyacrylamide gel electrophoresis (SDS-PAGE) was used^30^. The target band was subjected to mass spectroscopic analysis using LTQ-Orbitrap. To obtain the N-terminal sequence, we electroblotted the proteins on to a PVDF membrane as described in^31^ and the sequence was determined by using the method of Edman degradation^32^.

### Optimal reaction conditions

The enzyme assay was conducted at different temperatures to determine the optimum temperature. The 0.1 M Tris-HCl buffer (pH7.0) used in the enzyme assay was replaced by buffers at different pH values to alter the reaction pH for determination of optimum pH. This would provide a theoretic basis for the application of this NiR. Kinetic parameters were determined as follows: the concentration ranges of substrate (NaNO_2_) used ran from 1 mM to 5 mM, while other components of the NiR assay were as mentioned above. The values of Km and Vmax were obtained by linear plots of reciprocal velocities against the reciprocal of substrate concentration.

### The action of isolated NiR in removing nitrite *in vivo*

Excessive consumption of nitrite can lead to poisoning. In the experiment, 4-week-old male Kunming mice were used to test the effect of the isolated NiR on the survival of the intoxicated mice and the time required to sacrifice the mice. One hundred and twenty mice were divided into six groups with 20 mice per group. Food and water were withheld from the mice 12 hours before the experiment. Different doses of a solution of *B. edulis* NiR extracts were administered into the mice by intragastric administration. Mice in the control group received physiological saline instead. Three minutes later, sodium nitrite was given by intragastric administration. The durations of survival after treatment with low, intermediate and high doses of sodium nitrite were recorded. The duration of survival was used to examine the effect of the enzyme on eliminating sodium nitrite *in vivo*.

For determining the reduction in blood nitrite level caused by the NiR, male Sprague-Dawley rats weighing 200–250 g were used. A dose of 7 mg nitrite per 100 g body weight was introduced by intragastric administration. Three minutes later, the experimental group of rats received, by intragastric administration, a fixed amount of *B. edulis* NiR. The control group received the same volume of physiological saline instead. Blood samples were collected from the tail vein at different time intervals. After deproteination by means of chemicals (ZnSO_4_ and NaOH, and ultrafiltration were chosen^29^,^33^), the nitrite level was determined by using the Griess method. To overcome the problem of gastric digestion, we packaged this NiR powder in capsules which can only be absorbed by the intestine in order to prolong the half-life of this enzyme in the body. The rats in the experimental group received two capsules that contained a total amount of 40 U enzyme by intragastric administration, while the control group did not receive the enzyme. Both groups of rats were given sodium nitrite by intragastric administration repeatedly at an interval of 90 min. The determination of blood nitrite content was conducted as mentioned above except the blood samples were collected at 15 min intervals.

The animal experiments had the prior approval of the Animal Experimentation Ethics Committee of The Chinese University of Hong Kong (Ref no. 12/035/MIS). The animals were sacrificed under light ethyl ether anesthesia.

## Additional Information

**How to cite this article**: Zhang, W. *et al.* *Boletus edulis* Nitrite Reductase Reduces Nitrite Content of Pickles and Mitigates Intoxication in Nitrite-intoxicated Mice. *Sci. Rep.* **5**, 14907; doi: 10.1038/srep14907 (2015).

## Supplementary Material

Supplementary Information

## Figures and Tables

**Table 1:** Purification of nitrite reductase from *Boletus edulis* fruiting bodies.

	Protein (mg)	Total activity (U)	Specific activity (U/mg)	Purification fold	Yield (%)
Crude extract	29250.0	22229.6	0.8	1.0	100
Ammonium sulfate precipitation (25%–65%)	4823.0	8055.7	1.7	2.2	36.2
SP-Sepharose and then followed by Q-Sepharose	414.4	3040.5	7.3	9.7	13.7
Mono Q 4.6/100 PE	84.0	977.0	11.6	15.3	4.4
Superdex75	22.1	259.4	11.8	15.5	1.2

**Table 2:** Sequence homology between the sequenced inner peptides and the corresponding peptides of other nitrite reductases.

Accession number	Fungal species	Amino acid sequence of inner peptide
peptide 3		RSSFVNVKETRQ
ref|XP_001840498.2|	*Coprinopsis cinerea okayama*7#130	**FVN**TH**ETR**
emb|CAC41649.1|	*Ustilago maydis*	**FVN**TD**ET**
emb|CBQ68461.1|	*Sporisorium reilianum* SRZ2	**FVN**TD**ET**
emb|CCF50610.1|	*Ustilago hordei*	**FVN**TD**ET**
ref|XP_001887050.1|	*Laccaria bicolor* S238N-H82	**FV**GIK**E**
peptide 6		RMSAAQMRGLADISARF
gb|EYE91674.1|	*Aspergillus ruber* CBS 135680	**M**T**A**IK**QMR**A**LA**
gb|EGO54037.1|	*Neurospora tetrasperma* FGSC 2508	**M**SP**A**H-H**GL**Q**D**TND**RF**
gb|EMF15199.1|	*Sphaerulina musiva* SO2202	R**GL**Q**D**TND**RF**
peptide 7		DLEALIDVTEIPHPGR
gb|KDE09322.1|	*Microbotryum violaceum p1A1 Lamole*	**DLE**SIM**D**YA**ELPH**
ref|XP_001887050.1|	*Laccaria bicolor* S238N-H82	**DL**D**AL**M**D**
ref|XP_001840498.2|	*Coprinopsis cinerea okayama*7#130	**DL**D**AL**M**D**
emb|CDM28696.1|	*Penicillium roqueforti*	**DLE**R**LID**
peptide 4		RIATDAGIHVERGIVVDDQMRT
ref|XP_007368696.1|	*Dichomitus squalens* LYAD-421 SS1	**VERG**G**IVVDD**RL**RT**
gb|EJP65304.1|	*Beauveria bassiana*	**GIVVDD**G**MRT**

**Table 3:** Effect of *Boletus edulis* nitrite reductase (NiR) on duration of survival in nitrite-intoxicated mice.

Group number	Dosage of NiR (U/100 g body weight)	NaNO_2_dose (g/100 g body weight)	Mean survival time (min)	Prolongation of survival duration (%)
1	0	0.015	Mice all alive	—
2	0	0.025	18.1 ± 9.5	—
3	34.2	0.025	27.6 ± 5.32 mice alive	52.5
4	57	0.025	45.0 ± 15.012 mice alive	148.6
5	0	0.036	11.5 ± 3.7	—
6	57	0.036	17.0 ± 3.8	47.8

Results are presented as mean ± SD (n = 20).
