# Supplementary material for: Boletus edulis Nitrite Reductase Reduces Nitrite Content of Pickles and Mitigates Intoxication in Nitrite-intoxicated Mice
Source: Sci Rep. 2015 Oct 8;5:14907. doi: 10.1038/srep14907 (PMC4597360; doi:10.1038/srep14907)
Supplement: Supplementary Information [file srep14907-s1.doc]

***Boletus edulis* Nitrite reductase Reduces Nitrite Content of Pickles and Mitigates Intoxication in Nitrite-intoxicated Mice**

Weiwei Zhang ^a^, Guoting Tian^b^, Shanshan Feng ^a^ , Jack Ho Wong ^b^, Yongchang Zhao^b^, Xiao Chen ^a^, Hexiang Wang ^a^* , Tzi Bun Ng ^c^*

a State Key Laboratory for Agrobiotechnology and Department of Microbiology, China Agricultural University, Beijing 100193, China

b Institute of Biotechnology and Germplasmic Resource, Yunnan Academy of Agricultural Science, Kunming 650223, P. R. China

c School of Biomedical Sciences, Faculty of Medicine, The Chinese University of Hong Kong, Shatin, New Territories, Hong Kong, China

* Corresponding author. Tel./fax: 86 10 6273 2578.

E-mail addresses: hxwang1957@gmail.com (HX. Wang), e-mail: [b021770@mailserv.cuhk.edu.hk](mailto:b021770@mailserv.cuhk.edu.hk) (TB Ng)

Supplementary files

Table S1. Comparison of N-terminal sequences of *B. edulis* nitrite reductases with sequences of other fungal nitrite reductases.

| Fungal species | N-terminal sequence of nitrite reductase |
| --- | --- |
| *Boletus edulis* | **KPSPAQFPPLEWLGY** |
| *Serpula lacrymans var. lacrymans* S7.9] | **P**R**PA**DWPK**QFPP** |
| *Neurospora tetrasperma* FGSC 2508 | **PSP**PST**PP** |
| *Metarhizium anisopliae* ARSEF 23 | **PL**Q**W**AAQYDEASVS**LGY** |
| *Phanerochaete carnosa* HHB-10118-sp | **KP**A**PA**TSA**PL** |
| *Coprinopsis cinerea okayama*7#130 | **KPSP** |

**Table S2. Characteristics of *Boletus edulis* nitrite reductase (NiR) with isolated NiR from other species.**

| Species | Yield | Specific activity（U/mg） | Chromatographic characteristics | Molecular weight（kD） | Number of subunits | Optimum pH | Optimum temp |
| --- | --- | --- | --- | --- | --- | --- | --- |
| *Boletus edulis* | 1.2 | 11.8 | Unadsorbed on SP-sepharose，adsorbed on Q-Sepharose and mono-Q | 45 | 2 | 6.8 | 45 |
| *Haloferax mediterranei*[*^1^*](#_ENREF_1) | 4 | 15.79 | Adsorbed on Sepharose-4B, DEAE cellulose and Q-Sepharose | 66 | ND | 7.5 | 60 |
| *Desulfovibrio vulgaris*[*^2^*](#_ENREF_2) | ND | ND | Adsorbed on DEAE cellulose and Q-Sepharose | 56 and 18 | 2 | ND | ND |
| *Monoraphidium braunii*[*^3^*](#_ENREF_3) | 38 | 214.4 | Adsorbed on DEAE cellulose and Phenyl-Sepharose | 63 | ND | ND | ND |
| *Hydrogenobacter thermophilus*TK-6[^4^](#_ENREF_4) | 16 | 0.202 | Adsorbed on Phenyl Sepharose, Resource PHE and MonoS | 61.5 | Not  known | 6.5-7.0 | 70-75 |
| *Sinorhizobium meliloti*[*^5^*](#_ENREF_5) | ND | ND | Adsorbed on Source 15Q matrix column | 127 | 3 | 5 | ND |

“ND” means “No details available”.

reference

1 Martı́nez‐Espinosa, R. M., Marhuenda‐Egea, F. C. & Bonete, M. a. J. Purification and characterisation of a possible assimilatory nitrite reductase from the halophile archaeon Haloferax mediterranei. *FEMS Microbiology Letters* **196**, 113-118 (2001).

2 Pereira, I. A. C., LeGall, J., Xavier, A. V. & Teixeira, M. Characterization of a heme c nitrite reductase from a non-ammonifying microorganism, Desulfovibrio vulgaris Hildenborough. *Bba-Protein Struct M* **1481**, 119-130, doi:Doi 10.1016/S0167-4838(00)00111-4 (2000).

3 Vigara, J., Garcia-Sanchez, M. I., Garbayo, I., Vilchez, C. & Vega, J. M. Purification and characterization of ferredoxin-nitrite reductase from the eukaryotic microalga Monoraphidium braunii. *Plant Physiol Bioch* **40**, 401-405, doi:Pii S0981-9428(02)01385-2, Doi 10.1016/S0981-9428(02)01385-2 (2002).

4 Suzuki, M., Hirai, T., Arai, H., Ishii, M. & Igarashi, Y. Purification, characterization, and gene cloning of thermophilic cytochrome cd1 nitrite reductase from Hydrogenobacter thermophilus TK-6. *J Biosci Bioeng* **101**, 391-397, doi:10.1263/jbb.101.391 (2006).

5 Ferroni, F. M., Guerrero, S. A., Rizzi, A. C. & Brondino, C. D. Overexpression, purification, and biochemical and spectroscopic characterization of copper-containing nitrite reductase from Sinorhizobium meliloti 2011. Study of the interaction of the catalytic copper center with nitrite and NO. *J Inorg Biochem* **114**, 8-14, doi:DOI 10.1016/j.jinorgbio.2012.04.016 (2012).
